# Beyond B cell helpers: diverse roles of peripheral helper T cells

**DOI:** 10.1093/intimm/dxag025

**Published:** 2026-05-15

**Authors:** Yuki Masuo, Hiroyuki Yoshitomi

**Affiliations:** Department of Immunology, Graduate School of Medicine, Kyoto University, Kyoto 606-8501, Japan; Kyoto University Immunomonitoring Center, Kyoto University, Kyoto 606-8501, Japan; Department of Immunology, Graduate School of Medicine, Kyoto University, Kyoto 606-8501, Japan; Kyoto University Immunomonitoring Center, Kyoto University, Kyoto 606-8501, Japan; Institute for the Advanced Study of Human Biology, Kyoto University, Kyoto 606-8501, Japan

**Keywords:** autoimmunity, stem-like T cell population, tertiary lymphoid structure, tissue inflammation

## Abstract

Peripheral helper T cells (T_PH_ cells) constitute a subset of CD4^+^ T cells that provide help to B cells in inflamed non-lymphoid tissues. T_PH_ cells have been identified across various chronic disorders, especially autoimmune diseases, and accumulating evidence suggests their involvement in disease pathogenesis. However, the mechanisms by which T_PH_ cells drive persistent inflammation remain incompletely understood. Recent insights reveal that T_PH_ cells play additional roles in local immune responses beyond their classical B cell helper function. In this review, we explore the heterogeneity and functional diversity of T_PH_ cells that contribute to chronic inflammatory processes within target tissues of autoimmunity. These functions of T_PH_ cells include their stem-like capacity to serve as a reservoir and replenish activated cells *in situ,* as well as their direct role in the activation of macrophages through proinflammatory soluble factors. We further discuss the dynamic interplay between T_PH_ cells and tertiary lymphoid structures (TLSs), highlighting the chemokine regulation for TLS formation and the essential niche these structures offer for the T_PH_ cell maturation. A deeper understanding of T_PH_ cell-mediated tissue inflammation will pave the way for the development of novel therapeutic strategies in persistent autoimmune conditions.

## Teaser

Novel TPH cell functions in chronic inflammation

## Introduction

CD4^+^ helper T cells play a fundamental role in protective immune responses and maintain systemic homeostasis (1). A hallmark of their function is the interaction with B cells, which promotes B cell differentiation and antibody production (2, 3). However, dysregulation in this process can lead to autoantibody production in various autoimmune diseases (2, 3). Chronic autoimmune responses frequently induce the formation of functional immune cell aggregates containing T and B cells within inflamed tissues, called tertiary lymphoid structures (TLSs) (4). These TLSs share cellular and molecular features with secondary lymphoid organs (SLOs), including ongoing B cell maturation and antibody production (5). These observations have suggested that T–B cell interactions can occur within affected non-lymphoid tissues, thereby prompting efforts to identify T cell subsets that provide help to B cells in these tissues. As a result of a series of research, peripheral helper T (T_PH_) cells have gained attention as a B cell helper subset in non-lymphoid organs (6–8).

Early insights into T_PH_ cells were largely derived from studies in rheumatoid arthritis (RA), one of the most common autoimmune diseases characterized by synovial inflammation and joint destruction (9, 10). This is in part because RA provides a unique opportunity to obtain relatively large amounts of tissue samples for research through joint replacement surgery (11). Most RA synovial tissues show diffuse immune cell infiltration and/or the formation of TLSs, with more extensive accumulation observed in autoantibody-positive patients (12–14). Studies of CD4^+^ helper T cells within RA synovial tissues identified a subpopulation expressing the B cell chemoattractant CXCL13 (6, 7). Although CXCL13 production by follicular helper T (T_FH_) cells in SLOs had been well established (15, 16), the majority of these CD4^+^ CXCL13^+^ T cells in RA synovium lacked expression of Bcl6 (7, 8), the canonical transcription factor of T_FH_ cells (17–19). Phenotypically, these T cells showed high expression of PD-1 in the absence of CXCR5 (7, 8). Further functional experiments revealed that, similar to conventional T_FH_ cells, CD4^+^ PD-1^high^ CXCR5^neg^ T cells isolated from RA synovium induced B cell differentiation and antibody production in an IL-21 and SLAMF5-dependent manner (8). On the basis of the findings above, these B cell helpers in inflamed non-lymphoid tissues were considered to be distinct from T_FH_ cells observed in SLOs and are now recognized as T_PH_ cells (20–23).

Subsequent studies have demonstrated the presence of T_PH_ cells in the target tissues of multiple autoimmune diseases, including systemic lupus erythematosus (SLE) (24, 25), Sjögren’s syndrome (26, 27), systemic sclerosis (28), celiac disease (29), ulcerative colitis (30), and bullous pemphigoid (31). T_PH_ cells can also be found in the blood of patients with autoimmune diseases (22), and the frequency of circulating T_PH_ cells correlates with disease activity in seropositive RA (8, 32, 33), SLE (34–36), IgG4-related disease (37, 38), and IgA nephritis (39). These observations suggest that the emergence of T_PH_ cells is a component of pathological immune activation, and these cells may directly contribute to inflammation. However, a key question remains: how do T_PH_ cells drive chronic inflammation within affected tissues?

Emerging evidence, particularly from single-cell technologies, has revealed heterogeneity within T_PH_ cells, characterized by distinct gene expression signatures (14, 40, 41). Despite these insights, the functional properties of different subpopulations were largely unexplored. In this review, we highlight recent advances in understanding how distinct subsets of T_PH_ cells are functionally specialized and collectively contribute to persistent tissue inflammation. Specifically, we discuss the strategies for defining distinct T_PH_ subsets, the mechanisms underlying their maintenance within tissues, and their roles in inflammatory processes. We further discuss the link between T_PH_ cells and TLSs, revealed by advances in spatial transcriptomics that have enabled a more precise understanding of immune responses within tissues.

## Two distinct subsets of T_PH_ cells

The expression of chemokine receptors on T cells is an important factor in their migratory capacity (42, 43) and serves as a robust marker to define distinct T cell subsets (44). T_PH_ cells express a set of chemokine receptors that allow them to respond to inflammatory signals in tissues, such as CCR2, CCR5, and CX3CR1 (8). However, it has also been shown that a substantial proportion of T_PH_ cells in RA synovium retain the expression of CCR7 (6), a key molecule associated with naive and central memory phenotypes. This observation is further supported by single-cell RNA sequencing (scRNA-seq) data, which consistently identified CCR7^+^ T_PH_ cells (14, 41).

A recent study conducted a comprehensive profiling of chemokine receptor expression across T_PH_ subsets defined by scRNA-seq (45). Analysis of synovial CD4^+^ T cells from RA patients identified two transcriptionally distinct subpopulations of T_PH_ cells, with CCR7 and CXCR6 emerging as suitable candidates for distinguishing these subpopulations. Inflammatory receptors, including CCR2 and CCR5, were more prominently expressed in CXCR6^+^ T_PH_ cells. Consistent with the transcriptomic data, CCR7 and CXCR6 showed a mutually exclusive expression pattern on T_PH_ cells at the protein level, confirmed by flow cytometry, thereby enabling the isolation of CCR7^+^ T_PH_ and CXCR6^+^ T_PH_ cells for functional characterization.

An important question here is whether these T_PH_ subpopulations are also present in the peripheral blood, particularly in clinical settings where access to affected tissue samples is often limited. Transcriptomic profiling of blood CD4^+^ T cells from RA patients identified cells with similar gene expression signatures to both CCR7^+^ T_PH_ and CXCR6^+^ T_PH_ subsets (45), suggesting that these subsets can enter the circulation. In support of this, blood and tissue T_PH_ cells shared a part of their T-cell receptor (TCR) repertoire (41). Nonetheless, further studies are needed to identify markers accurately defining T_PH_ subpopulations in blood and to fully understand the relationship between blood and tissue T_PH_ cells.

## T_PH_ cells with stem cell features

CCR7^+^ T_PH_ and CXCR6^+^ T_PH_ cells in RA synovium introduced in the previous section shared core phenotypic features, including PD-1^high^ CXCR5^neg^ CXCL13^+^ expression. However, these subsets markedly differed in their gene expression signatures, especially those related to T-cell activation states and effector molecules (45). CCR7^+^ T_PH_ cells highly expressed *TCF7*, *LEF1*, and *IL7R*, genes related to T cell stemness (46, 47). By contrast, CXCR6^+^ T_PH_ cells upregulated genes involved in activated states and inflammatory processes, such as *HLA-DRB1*, *GZMA*, and *IFNG*. Open chromatin accessibility analyses further revealed that these two populations showed distinct epigenetic landscapes and transcription factor binding motifs. Taken together, CCR7^+^ and CXCR6^+^ T_PH_ cells represent stem-like and effector subsets, respectively.

Stem-like T_PH_ cells exhibited robust self-renewal capacity by showing rapid proliferation in response to TCR stimulation (45). The presence of Ki-67 expression among these cells in inflamed RA joints indicated active proliferation within tissues. This is consistent with previous findings showing that memory T cells with CCR7 expression possess greater proliferative potential compared to their CCR7-negative counterparts (48, 49). In addition to self-renewal, stem-like T_PH_ cells were demonstrated to serve as a progenitor pool for effector T_PH_ cells, as supported by multiple lines of evidence: (1) strong sharing of TCR clonotype repertoire between the two subsets, (2) computational trajectory inference based on transcriptomic data, and (3) successful *in vitro* differentiation of stem-like T_PH_ cells into effector T_PH_ cells. Thus, stem-like T_PH_ cells play an essential role in maintaining the overall T_PH_ population by continuously undergoing self-renewal and seeding activated effector cells within inflamed tissues.

## Stem-like T cell subsets in chronic disorders

The concept of a stem-like T cell subset serving as a persistent *in situ* reservoir extends to diverse T cell lineages across various contexts. Indeed, T cells with stem-like features have been described in a range of chronic conditions, such as chronic infections, malignancies, and autoimmunity (50–52). Early insights into these stem-like T cells were derived from mouse models of chronic viral infection, such as lymphocytic choriomeningitis virus (LCMV), where T cells typically acquire an exhausted phenotype (53). Unlike exhausted CD8^+^ T cells, which exhibit poor proliferative capacity and effector function, a stem-like subset called progenitors of exhausted T (T_PEX_) cells retains high proliferative potential and the ability to sustain the T cell response (54–56). T_PEX_ cells undergo self-renewal and differentiate into transitory cells; these cells exert effector functions to control the infection before eventually progressing into a terminally exhausted state (57), highlighting the pivotal role of T_PEX_ cells in the control of chronic viral infection by replenishing effector cells.

T_PEX_ cells have also been characterized in the field of cancer immunology, providing direct implications for clinical practice. Among tumor-infiltrating lymphocytes in humans, a subset of stem-like CD8^+^ T cells displays enhanced self-renewal and multipotency despite their exhausted phenotype (58). These intratumoral T_PEX_ cells express PD-1 and drive the proliferative burst of effector CD8^+^ T cells following PD-1 blockade (59, 60). Furthermore, a higher abundance of T_PEX_ cells within tumor lesions is a potent predictor of favorable responses to immune checkpoint blockade (61, 62), underpinning their role in determining the therapeutic efficacy of these treatments.

The proliferative burst of T_PEX_ cells is regulated by the transcription factor T cell factor 1 (TCF-1), encoded by *TCF7* (54–56, 59, 60). TCF-1 plays a dual role by maintaining T-cell stemness while restraining terminal differentiation towards a dysfunctional state (46). Notably, TCF-1 performs a functionally analogous role in stem-like T_PH_ cells in RA (45). Deletion of *TCF7* in these cells resulted in reduced proliferative capacity and accelerated differentiation into effector T_PH_ cells. Furthermore, *TCF7* has been identified as a susceptibility locus for multiple autoimmune diseases through genome-wide association studies (GWAS), including RA (63, 64), SLE (65–69), and multiple sclerosis (MS) (70–72), suggesting that TCF-1^+^ stem-like T cells may be involved in the pathogenesis of a broad spectrum of autoimmune conditions.

In line with this, recent studies both in mice and humans have identified stem-like T cells driving autoimmune pathogenesis (45, 73–78). These stem-like T cells give rise to diverse pathogenic subsets depending on the context, including T_H_17 cells in encephalomyelitis (73, 74), ulcerative colitis (77), and arthritis (78); T_FH_ cells and cytotoxic CD4^+^ T cells in vasculitis (76); and cytotoxic CD8^+^ T cells in type 1 diabetes (75) and ulcerative colitis (77). These findings indicate that stem-like T cells likely play a crucial role in sustaining chronic inflammation in autoimmunity by continuously replenishing the pathogenic T cell pool. Therefore, a deeper understanding of stem-like T-cell biology offers a promising therapeutic approach in chronic disorders. Inducing these populations can potentially enhance immune responses against chronic infections and cancer, whereas inhibition or depletion may provide a strategy for resolving persistent inflammation in autoimmune diseases.

## T_PH_ cells and TLSs

In target tissues of chronic inflammation, infiltrating immune cells frequently organize into specialized niches, or TLSs, that facilitate local cellular interactions. These TLSs exhibit considerable heterogeneity in their organization and cellular composition, and three stages of maturation have recently been proposed: (1) a least-organized stage composed of dense lymphocytic aggregates lacking both CD21^+^ follicular dendritic cells (FDCs) and clear segregation of T and B cell zones, (2) a primary follicle-like stage with the presence of FDCs but lacking germinal centers (GCs), and (3) a matured secondary follicle–like stage with both FDCs and GCs (79, 80). Here, TLSs encompass all stages of these aggregates described above, following recent nomenclature (79, 80).

Recent advances in spatial omics technologies have provided unprecedented opportunities to explore tissue organization at high resolution, leading to mechanistic insights into disease pathology *in situ* (81). Spatial omics allows for high-plex gene and protein expression profiling, facilitating the identification of diverse cell types along with their spatial distribution and cellular neighborhoods. In addition, specific tissue niches can be defined and systematically identified by integrating multiple parameters, such as cellular density, niche size, and cellular composition within these microenvironments.

A recent study applied spatial transcriptomics to RA synovial tissues and identified TLS regions based on cellular density and aggregate size (45). Within these TLSs, stem-like T_PH_ cells were abundantly found, expressed high levels of CXCL13, and colocalized with B cells. The functional significance of this colocalization was supported by *in vitro* assays, in which coculture of synovial stem-like T_PH_ cells and B cells induced the differentiation of stem-like T_PH_ cells into effector T_PH_ cells as well as the antibody production from B cells. These findings indicate that T-B interactions within TLSs contribute to activation and differentiation of both T and B cells. Notably, similar spatial characteristics of stem-like T cells have been observed in various chronic inflammatory conditions. In systemic sclerosis, CCR7^+^ CXCL13^+^ T cells accumulated within perivascular TLSs in lesional skin and positioned adjacent to B cells (28). Likewise, in the tumor microenvironment, T_PEX_ cells were identified as the predominant subset within TLSs among exhausted T cells (52). These observations suggest that TLSs provide a specialized niche for stem-like T cells to proliferate and differentiate at the site of inflammation.

CXCL13, produced by stem-like T_PH_ cells, likely serves as a critical driver for the formation of TLSs beyond its role in B cell recruitment. In RA synovium, T_PH_ cells represent the main source of CXCL13 (82), a chemokine whose ectopic expression was sufficient to induce TLS formation in mouse models (83). CXCL13 expression in human CD4^+^ T cells is regulated by multiple soluble factors such as TGF-β (84), type I interferon (85), and insulin-like growth factor-like family member 2 (IGFL2) (86) as well as the transcription factors MAF and SOX4 (34, 87). Notably, murine CD4^+^ T cells express negligible levels of CXCL13, if any, even under inflammatory conditions (3), illustrating the unique feature of human T_PH_ cells and highlighting the importance of investigating human samples to further elucidate the mechanisms of TLS formation.

How stem-like T_PH_ cells are initially generated and recruited to the synovium remains an open question. The recruitment of CCR7^+^ T cells to RA synovium appears to be mediated by fibroblast-like synoviocytes (FLSs) expressing CCL19 and CCL21, the ligands of CCR7 (45). These FLSs are positioned in perivascular regions, where they guide CCR7^+^ T cells from the blood circulation into the tissue. The presence of dendritic cells and naive T cells in RA synovium suggests that stem-like T_PH_ cells may undergo differentiation within the synovial tissues (88). Alternatively, stem-like T_PH_ cells may differentiate within SLOs prior to their recruitment to inflamed tissues. A deeper understanding of the origin of stem-like T_PH_ cells will provide insights into the initial steps of TLS formation and tissue inflammation. Additionally, given that T_PH_ cells can enter the bloodstream, these circulating cells may migrate to other joints. If such inter-articular migration occurs, not only stem-like T_PH_ cells but also effector T_PH_ cells from the circulation might play a role as drivers of TLS formation and tissue inflammation in the course of disease progression. This aligns with a previously proposed model in which T_PH_ cells expressing CCR2 and CCR5 home to inflamed tissue from the circulation (11).

Another fundamental question is how the balance between self-renewal and differentiation of stem-like T_PH_ cells is regulated within the TLS. We speculate that both disease activity and clinical stage, including early onset, active disease, and remission, influence this balance. Active disease is associated with a high frequency of effector T_PH_ cells (45, 86), suggesting that the balance shifts toward differentiation. When the inflammation progresses into a persistent phase, stem-like T_PH_ cells tend to accumulate (88). This may result from the enhanced self-renewal of existing stem-like T_PH_ cells and/or the potential conversion of effector cells into a memory state. Identifying the molecular switches that govern the balance between self-renewal and differentiation in stem-like T_PH_ cells will be crucial for a deeper understanding of disease progression.

## T_PH_ cells and tissue inflammation

As stated in the introduction, active involvement of T_PH_ cells in tissue inflammation has been suggested based on the observations that circulating T_PH_ cell frequencies correlate with disease activity in multiple autoimmune diseases. Thus, research has focused on revealing the molecular mechanisms by which T_PH_ cells drive local inflammation, leading to insights into proinflammatory soluble factors produced by T_PH_ cells, such as IFN-γ (89–91), GM-CSF (89, 92), and IGFL2 (86). The expression levels of these molecules are higher in effector T_PH_ cells than in the stem-like population (confirmed in our datasets), aligning with the correlation between disease activity and the frequency of effector T_PH_ cells in inflamed tissues in RA (45). These findings suggest that the effector T_PH_ cells may play an important role in tissue inflammation through these proinflammatory molecules.

In contrast to stem-like T_PH_ cells localized within TLSs, effector T_PH_ cells were mainly found outside of TLSs in RA synovium, where they were proximal to macrophages and CD8^+^ T cells (45). Emerging lines of evidence suggest that effector T_PH_ cells interact with and activate monocytes/macrophages, leading to tissue inflammation driven by proinflammatory macrophages. One example of the molecules mediating this crosstalk is IFN-γ, which promoted the polarization of macrophages into a proinflammatory state and triggered the secretion of cytokines such as tumor necrosis factor-alpha (TNF-α) (93). Another example is GM-CSF, which was shown to drive the differentiation of monocytes into CD1c^+^ dendritic cells *in vitro* (92). These CD1c^+^ dendritic cells were enriched within inflamed joints (14, 92), suggesting the potential role for GM-CSF in inducing an inflammatory myeloid subset.

A recent study identified IGFL2, a member of the IGFL family, as a novel soluble factor that mediates the interaction between effector T_PH_ cells and macrophages in RA (86). Although IGFL2 shares structural features with the insulin-like growth factor (IGF) family, it does not bind to IGF receptors; instead, it binds to IGFLR1, which is its only known receptor to date (94, 95). The expression of IGFL2 within RA synovium was specific to T_PH_ cells, while IGFLR1 was broadly expressed by multiple immune cell subsets, including macrophages. Mechanistically, IGFL2 stimulation of monocytes activated the NF-κB pathway and induced the upregulation of genes related to the type II interferon pathway, including CXCL9, MX1, ISG15, and IFITMs. This was consistent with the structural properties of IGFLR1, which possesses an intracellular domain similar to those of the TNF receptor superfamily (95). Notably, the gene signature induced by IGFL2 was observed in proinflammatory macrophages within RA synovium and was significantly enriched within patients with active disease compared to those in remission. In addition, circulating IGFL2 levels measured in blood correlated with disease activity, further supporting the involvement of IGFL2 in inflammation. Together, these data indicate that T_PH_ cells play a critical role in shifting macrophages into a pathogenic state through IGFL2 production. Additionally, as IGFL2 is only present in primates, IGFL2 provides an exemplar of how genomic traits acquired during human evolution can drive autoimmune pathogenesis (96).

The functional properties of effector T_PH_ cells discussed above suggest that these cells can exert potent effector functions despite their expression of inhibitory molecules, such as PD-1, CTLA-4, and LAG-3. Apart from cytokines, effector T_PH_ cells produce cytotoxic molecules, including perforin and granzymes (14, 40, 45). Whether these molecules also contribute to tissue inflammation and/or damage should be elucidated in future studies. Furthermore, it remains to be investigated if effector T_PH_ cells function uniformly across diverse pathological conditions. The features and effector functions of these cells may be tuned during their *in situ* differentiation by the specific microenvironment of the inflamed tissue, potentially leading to distinct effector signatures across diseases.

## Concluding remarks

**Figure 1.**
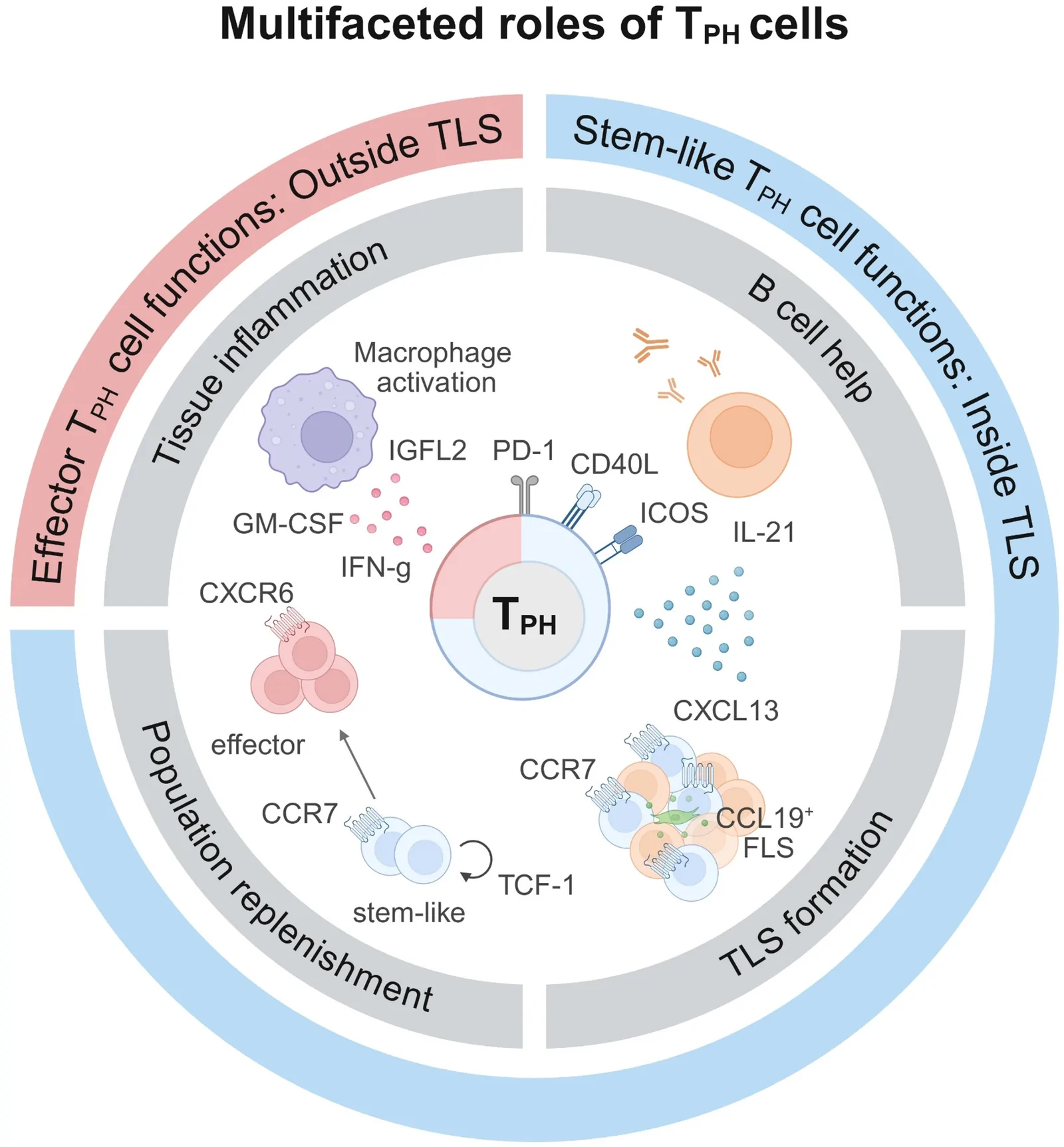
Heterogeneity and multifaceted roles of T_PH_ cells. T_PH_ cells highly express PD-1 and consist of distinct stem-like and effector subsets. Stem-like T_PH_ cells provide help to B cells through IL-21 secretion as well as CD40 ligand and ICOS expression. CCL19^+^ fibroblast-like synoviocytes (FLSs) likely recruit CCR7^+^ stem-like T_PH_ cells, which secrete CXCL13 to promote TLS formation. Stem-like T_PH_ cells express TCF-1 and maintain T_PH_ population through self-renewal and differentiation into their effector counterparts. In contrast, CXCR6^+^ effector T_PH_ cells drive tissue inflammation by activating macrophages through proinflammatory cytokines, including IGFL2, IFN-γ, and GM-CSF.

The studies discussed in this review have extended our understanding of T_PH_ cell functions beyond the classical framework of B cell help (Fig. 1). First, through soluble factors, including IGFL2, T_PH_ cells directly act on macrophages and induce their activation. This interaction between T_PH_ cells and macrophages provides a mechanistic insight into how T_PH_ cells contribute to tissue inflammation. Second, these proinflammatory effector T_PH_ cells can be continuously replenished from the stem-like subset within the inflamed tissues. By balancing self-renewal and differentiation, stem-like T_PH_ cells serve as a reservoir of the T_PH_ cell population *in situ*. TLSs offer a key microenvironment for this process, facilitating the crosstalk between stem-like T_PH_ and B cells. In addition, CXCL13 secretion and CCR7 expression by stem-like T_PH_ cells play important roles in the formation of TLSs.

In summary, T_PH_ cells acquire specialized functional roles at different stages of their differentiation. Functional diversity along differentiation is not unique to T_PH_ cells but is a feature shared with other T cell subsets. In particular, it parallels the well-described heterogeneity of T_FH_ cells, which encompass distinct stem-like memory, effector, and regulatory subsets (97, 98).

These insights into T_PH_ cell biology have opened up possibilities for therapeutic intervention. Given their capacity to sustain the local T_PH_ population, stem-like T_PH_ cells represent a promising therapeutic target. Elucidating the molecular basis of their self-renewal and differentiation processes will be critical for the development of such strategies. Additionally, targeting effector molecules, such as IGFL2, offers an alternative approach aimed at disrupting T_PH_ cell-mediated inflammation. Together, these therapeutic strategies have the potential to control persistent tissue inflammation and provide novel treatment options for patients with refractory disease.
